# The dual action of poly(ADP-ribose) polymerase -1 (PARP-1) inhibition in HIV-1 infection: HIV-1 LTR inhibition and diminution in Rho GTPase activity

**DOI:** 10.3389/fmicb.2015.00878

**Published:** 2015-08-25

**Authors:** Slava Rom, Nancy L. Reichenbach, Holly Dykstra, Yuri Persidsky

**Affiliations:** Department of Pathology and Laboratory Medicine, Temple University School of MedicinePhiladelphia, PA, USA

**Keywords:** HIV-1, PARP, LTR, NFκB, NeuroAIDS

## Abstract

Multifactorial mechanisms comprising countless cellular factors and virus-encoded transactivators regulate the transcription of HIV-1 (HIV). Since poly(ADP-ribose) polymerase 1 (PARP-1) regulates numerous genes through its interaction with various transcription factors, inhibition of PARP-1 has surfaced recently as a powerful anti-inflammatory tool. We suggest a novel tactic to diminish HIV replication via PARP-1 inhibition in an *in vitro* model system, exploiting human primary monocyte-derived macrophages (MDM). PARP-1 inhibition was capable to lessen HIV replication in MDM by 60–80% after 7 days infection. Tat, tumor necrosis factor α (TNFα), and phorbol 12-myristate 13-acetate (PMA) are known triggers of the Long Terminal Repeat (LTR), which can switch virus replication. Tat overexpression in MDM transfected with an LTR reporter plasmid resulted in a 4.2-fold increase in LTR activation; PARP inhibition caused 70% reduction of LTR activity. LTR activity, which increased 3-fold after PMA or TNFα treatment, was reduced by PARP inhibition (by 85–95%). PARP inhibition in MDM exhibited 90% diminution in NFκB activity (known to mediate TNFα- and PMA-induced HIV LTR activation). Cytoskeleton rearrangements are important in effective HIV-1 infection. PARP inactivation reduced actin cytoskeleton rearrangements by affecting Rho GTPase machinery. These discoveries suggest that inactivation of PARP suppresses HIV replication in MDM by via attenuation of LTR activation, NFκB suppression and its effects on the cytoskeleton. PARP appears to be essential for HIV replication and its inhibition may provide an effective approach to management of HIV infection.

## Introduction

Human immunodeficiency virus (HIV)-1 crosses the blood brain barrier and infects glial cells of the central nervous system (CNS). Human immunodeficiency virus (HIV)-1-associated neurological disorder (HAND) is defined as cognitive, motor and/or behavioral impairments caused by HIV replication, immune activation and release of neurotoxins in the brain that results in neuronal injury (Ellis et al., 2011). Even though under antiretroviral therapy some individuals demonstrate immune recovery, HAND incidence remains high and causes for that are not understood (Ellis et al., 2011). One current theory is that the neurodegeneration associated with HIV-1 infection is caused by chronic brain inflammation, concomitant with a low level of HIV-1 replication in CNS resident macrophages and microglia (Kraft-Terry et al., 2010; Thompson et al., 2011). Therefore, specific tactics aiming at inflammation and HIV-1 replication should be valuable for HAND amelioration.

Recently, poly(ADP-ribose) polymerase (PARP)-1 inhibitors have arisen as powerful immunomodulatory and anti-inflammatory compounds. PARP-1 (further in the text referred to as PARP), being a member of a family of NAD-dependent enzymes, produces approximately 80% of poly(ADP-ribose) (PAR) in the cell (Rouleau et al., 2010). Post-translational modification of proteins by poly(ADP-ribosyl)ation has well-known effects on various cellular functions involving cell death, differentiation and gene expression (Krishnakumar and Kraus, 2010). PARP, being an enzyme and a scaffold protein, is a crucial factor of the transcriptional machinery that regulates spatial organization of transcription-regulating supramolecular complexes, architecture of chromatin and histone shuttling (Krishnakumar and Kraus, 2010), thereby regulating inflammation-associated genes. The anti-inflammatory effects of PARP deactivation has been recently validated in animal models of meningitis, stroke, traumatic brain injury and multiple sclerosis (MS) (Lenzsér et al., 2007; Farez et al., 2009; Lescot et al., 2010; Rom et al., 2015), where inhibition of PARP diminished brain edema and decreased expression of adhesion molecules, infiltration of leukocytes and neuroinflammation (Zhang et al., 2004; Lenzsér et al., 2007). Previously, PARP inactivation was shown to play a role in the HIV-1 virus cycle, where PARP participated in HIV-1 LTR functions in U1 cells (Kameoka et al., 1999b, 2005), competed for binding to TAR RNA with positive transcription elongation factor b (p-TEFb) *in vitro* (Parent et al., 2005), and was required for efficient HIV-1 integration in (PARP-1^−/−^) mouse embryonic fibroblasts (Ha et al., 2001).

For the first time, in this study, we illustrate mechanisms of the modulatory effects of PARP inhibition on HIV-1 infection in primary human monocyte-derived macrophages (MDM). Our results suggest that PARP inactivation has a dual action in its effects on HIV-1 by negative regulation of HIV-1 LTR activity via NFκB inactivation and repressing actin cytoskeleton machinery through effects on the activity of small Rho GTPases. Both actin rearrangements and LTR are critical for HIV-1 infection and transfer of genetic material to the nucleus as well as HIV-1 gene transcription.

## Materials and methods

### Reagents and cells

The following PARP inhibitors were used: 5-aminoisoquinolinone (AIQ) and [N-(6-oxo-5,6-dihydro-phenanthridin-2-yl)-N,N-dimethylacetamide hydrochloride (PJ34) purchased from Enzo Life Sciences (Farmingdale, NY); 3-aminobenzamide (ABA), acquired from Sigma-Aldrich (St. Louis, MO), 3H-pyrido[4,3,2-de]phthalazin-3-one, 5-fluoro-8-(4-fluorophenyl)-2,7,8,9-tetrahydro-9-(1-methyl-1H-1,2,4-triazol-5-yl)-, (8S,9R)- (BMN 673, Talazoparib, BMN) and AZD2281 (AZD, Olaparib) purchased from Selleck Chemicals (Houston, TX), and 5′-deoxy-5′-[4-[2-[(2,3-dihydro-1-oxo-1H-isoindol-4-yl)amino]-2-oxoethyl]-1-piperazinyl]-5′-oxoadenosine dihydrochloride (EB47) purchased from Tocris Bioscience (Minneapolis, MN). PARP inhibitors were used in concentrations (ABA at 2 mM, PJ34, AZD, and EB47 at 10 μM, AIQ at 2 μM, and BMN at 20 nM) for the period of time indicated in the figure legends, and did not show any toxicity (Supplementary Figure 1). Recombinant human TNFα and PMA were purchased from R&D Systems (Minneapolis, MN) and Sigma-Aldrich, respectively. Rho A GTPase-specific inhibitor, CT04, and Rho GTPase switch™ activator, CN04, were purchased from Cytoskeleton Inc. (Denver, CO). Rac1 GTPase-specific inhibitor, NSC23766 (NSC), was purchased from EMD Chemicals (San Diego, CA).

Primary human monocytes from HIV-1 and hepatitis B seronegative donors were obtained from the University of Nebraska Medical Center, Department of Pharmacology and Experimental Neuroscience, Omaha, NE, after leukopheresis and were purified by countercurrent centrifugal elutriation (Ramirez et al., 2008). Monocytes (1 × 10^6^/well) were seeded into 24-well CellBind multiwell plates (Corning) within 24 h of isolation and maintained in Dulbecco's modified essential medium, high glucose (DMEM, Life Technologies, Carlsbad, CA) supplemented with 10% heat-inactivated fetal bovine serum (FBS), 100 U/ml penicillin, 100 μg/ml streptomycin, 1% non-essential amino acids, and 1% glutamine (2 mM) (complete medium) and incubated for 7 days (Ramirez et al., 2013; Persidsky et al., 2015). Culture medium was changed every 3 days. After 7 days in suspension culture, MDM were infected with three strains of HIV-1, two macrophage tropic strains (M-tropic) HIV-1_ADA_ (Ebenbichler et al., 1995) and HIV-1_JRFL_ (Naif et al., 2002), or a dual-tropic HIV-1_89.6_ strain (Yi et al., 1999) (obtained from the AIDS Research and Reference Reagent Program, Division of AIDS, NIAID, National Institutes of Health, Germantown, MD) at an m.o.i. of 0.1 infectious virus particles/target cell. MDM were either pre-treated with test compounds for 24 h or post-treated immediately after infection with HIV-1. After 4 h HIV-1 infection, MDM were gently washed with complete medium and treatments were replenished. MDM were then incubated, with half medium replacement on days 3, 5, and 7 post-HIV infection. Test compounds were replenished every 12 h. On day 7 post-infection, culture medium was collected and stored at −80°C until assay for HIV-1 reverse transcriptase (RT) activity. Data are presented as means of at least triplicate determinations (± SEM).

TZM-bl cells (reporter cell line) were obtained from the NIH AIDS Research and Reference Reagent Program. TZM-bl cells were grown in 6-well plates in DMEM containing 10% FBS, 100 units/ml penicillin, and 100 μg/ml streptomycin. All cell culture reagents (media, antibiotics, etc.) were purchased from Life Technologies.

### Plasmids and MDM transfection

The HIV-1 LTR-luciferase reporter plasmid and its deletion mutants were created by PCR and cloned into KpnI/BglII of pGL3 as described previously (Rom et al., 2010). The pEYFP-Tat101 fusion construct was cloned into the pEYFP-C1 plasmid (Clontech, Mountain View, CA) as described (Rom et al., 2011). Ready-To-Glow™ NFκB Secreted Luciferase Reporter plasmid was purchased from Clontech. All transfection assays were performed using the Lipofectamine LTX kit (Life Technologies). The pYFP-C1 plasmid was used as an internal control for transfection efficiency.

### Reverse transcriptase assay

HIV reverse transcriptase (RT) activity was determined in HIV-1 infected MDM cultures collected 3, 5, and 7 days after HIV infection. The assay was performed as described (Ramirez et al., 2013).

### Enzyme immunoassay for the detection of HIV-1 p24

HIV-1 p24 antigen capture assay (Advanced Bioscience Laboratories, Inc., Rockville, MD) was measured in tissue culture supernatants by double antibody sandwich enzyme immunoassay according to the manufacturer's directions.

### Single round HIV-1 infection and β-galactosidase assay

Recombinant luciferase-encoding HIV-1 virions were pseudotyped with the envelope from HIV-1_ADA_ as previously described (Guo et al., 2002). The pseudotyped virus was used for single round infectivity assays in TZM-bl cells with the β-galactosidase staining assay (Life Technologies). The presence of LTR-driven β-gal provides a measure of LTR activity. TZM-bl cells were infected with pseudotyped virus for 48 h in the presence of AIQ, PJ34, or AZT. Images of stained cells were obtained with an AxioCam HR camera attached to an Axio Observer Z1 microscope (Carl Zeiss MicroImaging, Thornwood, NY). AxioVision (v4.7) imaging software (Carl Zeiss MicroImaging) was used to analyze the images. The number of stained cells was enumerated as an average of triplicates for each condition from two independent experiments.

### Luciferase assays

For HIV-1 LTR activity, MDM were transfected with HIV-1 LTR-luciferase reporter plasmid and then stimulated with TNFα or PMA for 24 h. Alternatively MDM were co-transfected with pEYFP-Tat101 plasmid or pYFP-C1 (as a control) and 24 h later were harvested. Luciferase activity was measured in protein extracts with the dual-luciferase reporter assay system (Promega, Madison, WI). MDM were pretreated with or without PARP inhibitors (PARPi) for 1 h prior to stimulation with TNFα or PMA. Alternatively PARPi were added to the medium 3 h post-transfection with pEYFP-Tat101 plasmid. For NFκB activity, the plasmids, Ready-To-Glow™ NFκB Secreted Luciferase Reporter plasmid or Ready-To-Glow™ Secreted Luciferase Reporter plasmid, that do not have NFκB binding sites as a control, were transfected into MDM as described above. MDM were pretreated with or without PARPi for 1 h prior to stimulation with TNFα or PMA for 24 h; medium was collected and analyzed utilizing Ready-To-Glow Secreted Luciferase Assay (Clontech, Mountain View, CA) based on the secreted *Metridia* luciferase reporter according to the manufacturer's instructions.

### CXCR4 ligation and cross-linking

For receptor engagement studies, MDM were suspended in DMEM/1% FBS/L-glutamine at 5 × 10^6^ cells/ml. Surface CXCR4 antigen was ligated with monoclonal antibody (12G5, eBioscience Inc., San Diego, CA) at 4°C for 25 min at 15 μg/ml, washed twice in cold DMEM, and then cross-linked with 5 μg/ml goat anti-mouse F(ab′)_2_ (Thermo Scientific, Rockford, IL) for 25 min at 4°C. Cells were washed twice in cold DMEM, re-suspended in DMEM/1% FCS/L-glutamine and incubated at 37°C, 5% CO_2_ for times ranging from 0 min to 1 h. Reactions were stopped by placing the cells on ice. In inhibition studies, MDMs were pre-incubated with PARPi overnight.

### Small rho guanosine triphosphatase (GTPase) activity assay

RhoA and Rac1 GTPase activity was measured in unstimulated (basal) or stimulated primary MDMs (with/out pretreatment with PARPi) by cross-linking CXCR4 receptor with antibody or alternatively stimulated with the GTPase activator CN04 (1 μg/ml) (Cytoskeleton, Inc.), which served as a positive control (Yamamoto et al., 2008). To quantify RhoA and Rac1 GTPase activity, a commercially available G-LISA RhoA Activation Assay kit and G-LISA Rac1 Activation Assay kit (Cytoskeleton Inc., Denver, CO) were used.

### Flow cytometry

MDM were untreated or pre-treated with PARPi or Rac1/RhoA inhibitors for 1 h prior to stimulation for 5 min by adsorption with HIV-1_ADA_. As a positive control, cells were stimulated with the GTPase activator CN04 (1 μg/ml) (Cytoskeleton, Inc.). Cells were washed and fixed for 10 min with 4% methanol-free formaldehyde (Thermo Scientific, Rockford, IL). Cells were stained overnight in 1X Permeability Buffer (eBioscience Inc.) diluted in Flow Buffer (BD Biosciences, San Jose, CA) with antibodies against phosphorylated-cofilin (Cell Signaling Technology, Inc., Danvers, MA) and total cofilin-APC (Novus Biologicals, Littleton, CO). Following primary antibody incubation, the cells were washed and incubated in flow buffer containing isotype control antibody (BD Biosciences) or fluorophore-conjugated secondary antibody (eBioscience Inc.). A FACS BD Canto II flow cytometer (BD Biosciences) and FlowJo software were used to obtain and analyze data (Tree Star, Inc., Ashland, OR). Data were collected from at least 10,000 events and repeated for MDM from three different donors.

### Quantification of F-actin and G-actin

MDM were treated with PARPi for 1 h prior to HIV-1_ADA_ adsorption for 5 min. Cells were washed with ice-cold PBS and fixed for 10 min as described above. Filamentous actin (F-actin) was stained by Acti-stain™ 488 phalloidin (Cytoskeleton) and globular actin (G-actin) was stained with DNase 1-Alexa 591 (Life Technologies) according to the manufacturer's instructions. A FACS BD Aria flow cytometer (BD Biosciences) and FlowJo software were used to obtain and analyze data. The ratio of F-actin to G-actin was calculated by dividing mean fluorescent intensity (MFI) of F-actin by the MFI of G-actin in a cell population. Data were collected from at least 20,000 events and repeated twice with MDM from different donors.

### Viability assay

MDM were treated with PARPi for 24 h. Cells were washed with PBS and viability was performed using the Live-Dead assay (Life Technologies), according to the manufacturer's recommendations.

### Statistical analysis

Data are expressed as the mean ± SEM of experiments conducted multiple times. One-Way analysis of variance with Dunnet's *post-hoc* tests was used for multiple group comparisons (luciferase, FACS, HIV-1 RT assays). Prism v6.0c software (GraphPad Software Inc., San Diego, CA) was used for statistical analyses. A *p*-value of *p* < 0.05 was considered significant.

## Results

### PARP inhibition diminishes HIV-1 infection of MDM

**Figure 1:**
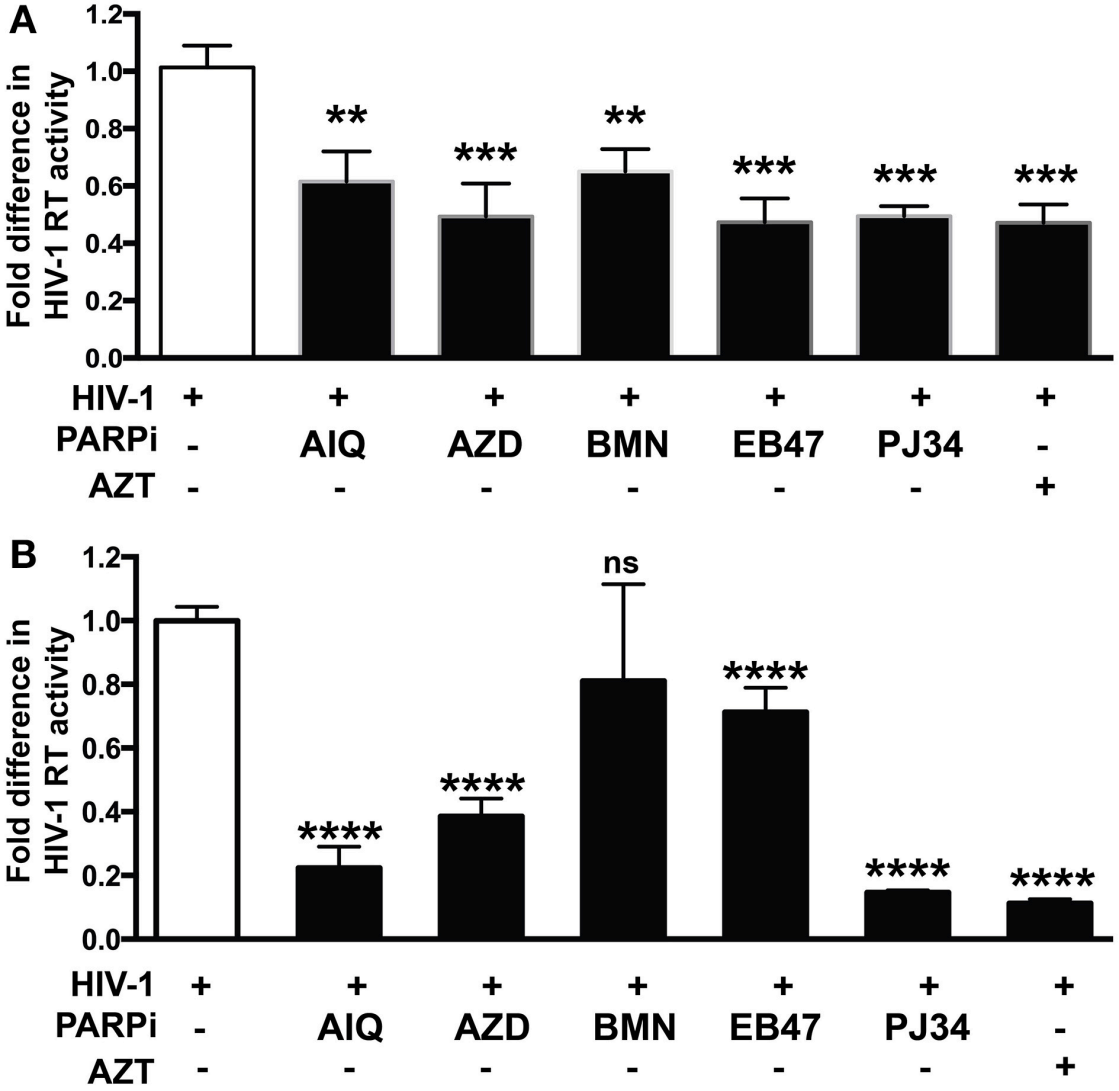
**PARP inhibition decreases HIV-1 reverse transcriptase (RT) activity and HIV-1 LTR activity in HIV-1 infected human macrophages (MDM)**. RT activity assays performed on HIV-infected MDM treated with PARPi or AZT either for 24 h prior to HIV infection **(A)** or immediately after **(B)** HIV infection. Treatments with PARP inhibitors (PARPi) or AZT were replenished every 12 h. The results are shown as the mean value ± SEM from three independent experiments utilizing MDM prepared from different donors. Data are presented as percent of HIV-only control. (^**^p = 0.05, ^***^p = 0.005, ^****^p < 0.0001, ns = not significant).

**Figure 2:**
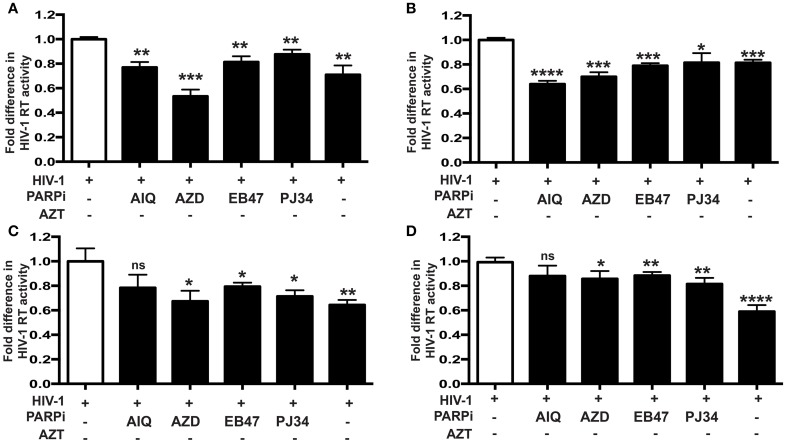
**PARP inhibition reduces HIV-1 reverse transcriptase (RT) activity and HIV-1 LTR activity in human MDM infected with different tropism HIV-1 strains**. RT activity assays performed on HIV-infected MDM treated with PARPi or AZT either for 24 h prior to HIV infection **(A,C)** or immediately after **(B,D)** HIV infection. MDM were infected with M-tropic JRFL strain **(A,B)** or dual-tropic strain 89.6 **(C,D)**. Treatments with PARP inhibitors (PARPi) or AZT were replenished every 24 h. The results are shown as the mean value ± SEM from three independent experiments utilizing MDM prepared from different donors. Data are presented as the percent of HIV-only control. (^*^p = 0.05, ^**^p = 0.01, ^***^p = 0.005, ^****^p < 0.0001, ns, not significant).

**Figure 3:**
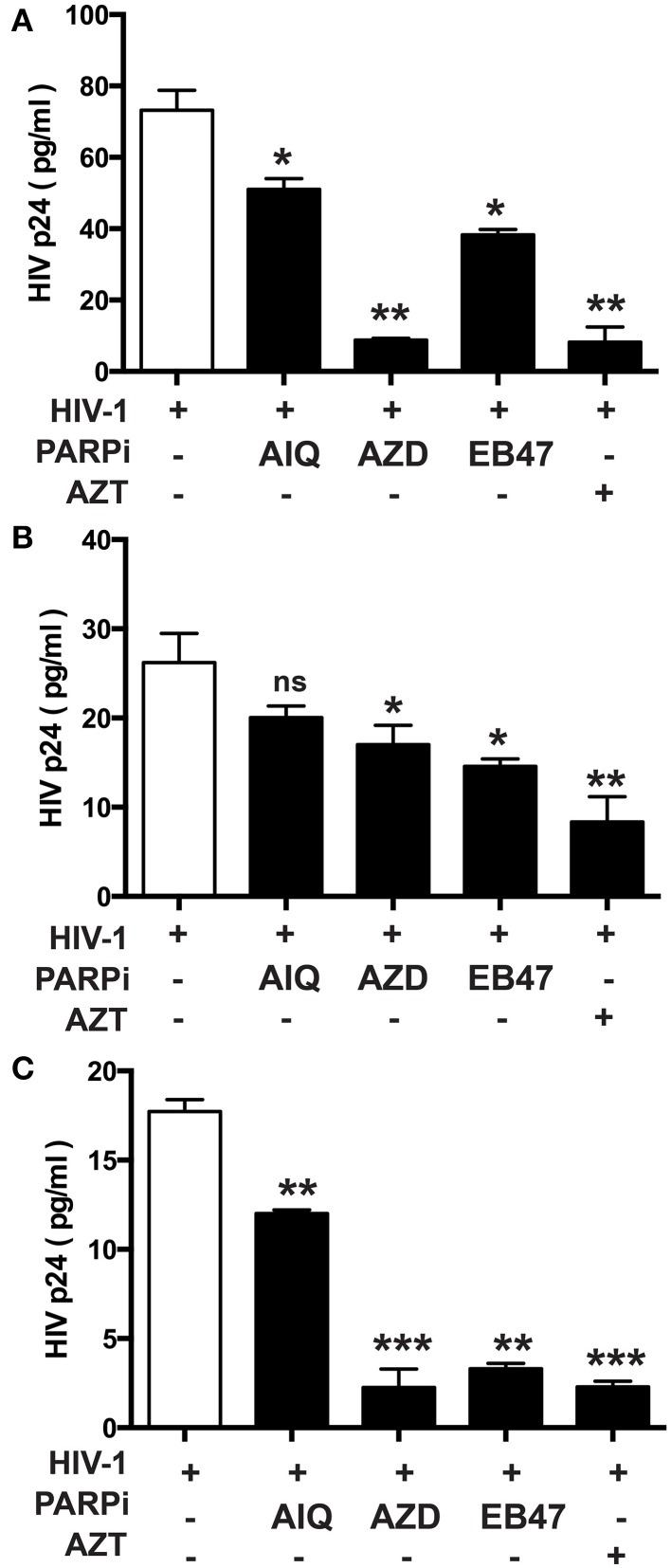
**Inhibition of PARP diminishes secretion of p24 HIV-1 gag protein in human MDM infected with different HIV-1 strains**. p24 ELISA was performed on supernatants from HIV-infected MDM treated with PARPi or AZT for 24 h prior to HIV infection. MDM were infected with M-tropic strains ADA **(A)** and JRFL **(B)** or the dual-tropic strain 89.6 **(C)**. Treatments with PARP inhibitors (PARPi) or AZT were replenished every 24 h. The results are shown as the mean value ± SEM from three independent experiments utilizing MDM prepared from different donors. Data are presented as percent of HIV-only control. (^*^p = 0.05, ^**^p = 0.005, ^***^p < 0.001, ns, not significant).

Given the gene regulatory role that PARP suppression plays in immune cells, we examined whether specific PARP inhibitors may affect HIV-1 replication. Differentiated MDM were infected with three strains of HIV-1: two M-tropic, HIV-1_ADA_ (Ebenbichler et al., 1995) and HIV-1_JRFL_ (Naif et al., 2002) and one dual-tropic (HIV-1_89.6_) (Yi et al., 1999) for 4 h; HIV-1 RT activity was measured after 7 days (as described in Materials and Methods). MDM were treated prior to HIV-1 infection or 4 h after HIV-1 infection with the PARP inhibitors as specified in Figure 1. Pretreatment with the PARPi AZD, EB47, and PJ34 resulted in 50 ± 3% reduction in HIV-1_ADA_ RT activity in MDM, whereas AIQ and BMN led to 40 ± 5% decrease (Figure 1A). When PARPi were added after HIV-1_ADA_ infection, AIQ and PJ34 diminished HIV-1 RT activity by almost 80%, while AZD and EB47 treatment resulted in 60 ± 2% and 25 ± 4% reduction in HIV-1 RT activity, respectively (Figure 1B). Of note, BMN treatment did not produce significant diminution in RT activity. The RT inhibitor, AZT (10 μM), significantly attenuated RT activity when added both prior to infection and after infection (Figures 1A,B). Pretreatment with the PARPi AZD resulted in 48 ± 3% reduction in HIV-1_JRFL_ or HIV-1_89.6_ RT activity in MDM, whereas EB47 and PJ34 led to a 20 ± 2% decrease (Figures 2A,C). When PARPi were added after HIV-1_JRFL_ or HIV-1_89.6_ infection, AZD, EB47, and PJ34 diminished HIV-1 RT activity by 20–30%, while AIQ treatment resulted in 35 ± 2% reduction in HIV-1_JRFL_ RT activity and did not produce significant diminution in HIV-1_89.6_ RT activity, respectively (Figures 2B,D). Inhibition of PARP in MDM resulted in a significant reduction in secretion of p24 HIV-1 gag protein in all three strains (Figure 3). Since PARP inhibition was not strain-specific, HIV-1_ADA_ was used in succeeding experiments. These results support the possible use of PARPi as potential anti-HIV-1 therapy.

### Inhibition of small rho GTPases affects HIV-1 infection

**Figure 4:**
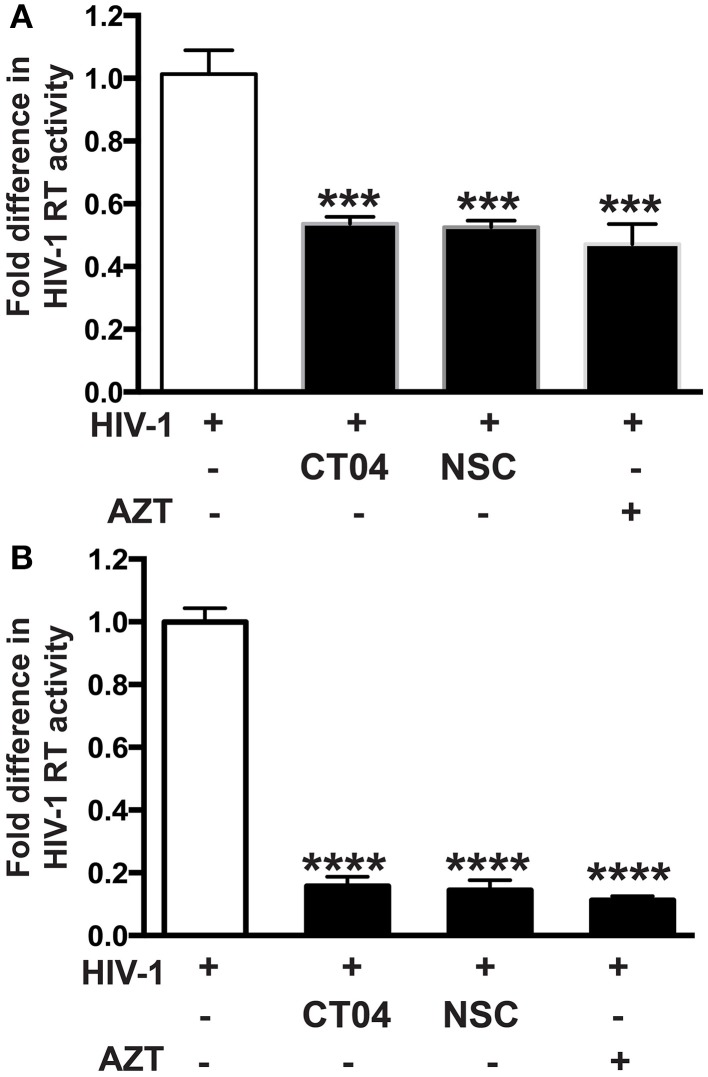
**Inhibition of Rho or Rac GTPase decreases HIV-1 reverse transcriptase (RT) activity in HIV-1 infected human macrophages (MDM)**. RT activity assays performed on HIV-infected MDM treated with Rho and Rac GTPase inhibitors, CT04, NSC, or AZT either for 24 h prior to HIV infection **(A)** or immediately after **(B)** HIV infection. The results are shown as the mean value ± SEM from three independent experiments utilizing MDM prepared from different donors. Treatments with Rho and Rac GTPase inhibitors or AZT were replenished every 12 h. (^***^p = 0.005, ^****^p < 0.0001).

Our group recently demonstrated that PARP inhibition suppresses activation of small Rho GTPases (Rom et al., 2015). Since small Rho GTPases are important in HIV-1 infection in different cell lines (Wang et al., 2000; Zoughlami et al., 2012), we decided to investigate whether small Rho GTPase inhibition would affect HIV-1 infection in primary MDM. Differentiated MDM were infected for 4 h and treated 1 h prior to or 4 h after HIV-1 infection with CT04 (1 μg/ml) and NSC (75 μM), RhoA or Rac1 GTPase inhibitors, respectively. HIV-1 RT activity was measured after 7 days infection. Inhibition of both RhoA and Rac1 GTPases resulted in 48 ± 2% decrease in HIV-1 RT activity, when inhibitors were applied prior to HIV-1 infection of MDM (Figure 4A), whereas post-infection treatment with Rho GTPase inhibitors led to 85 ± 3% diminution in HIV-1 RT (Figure 4B). The degree of inhibition in HIV-1 RT activity by small Rho GTPase inhibitors correlates with the extent of the effect of PARPi (Figure 1). These data support a connection between a role for PARP inhibition and GTPase activation in HIV-1 infection.

### Small GTPase activation is diminished in MDM upon PARP inhibition

**Figure 5:**
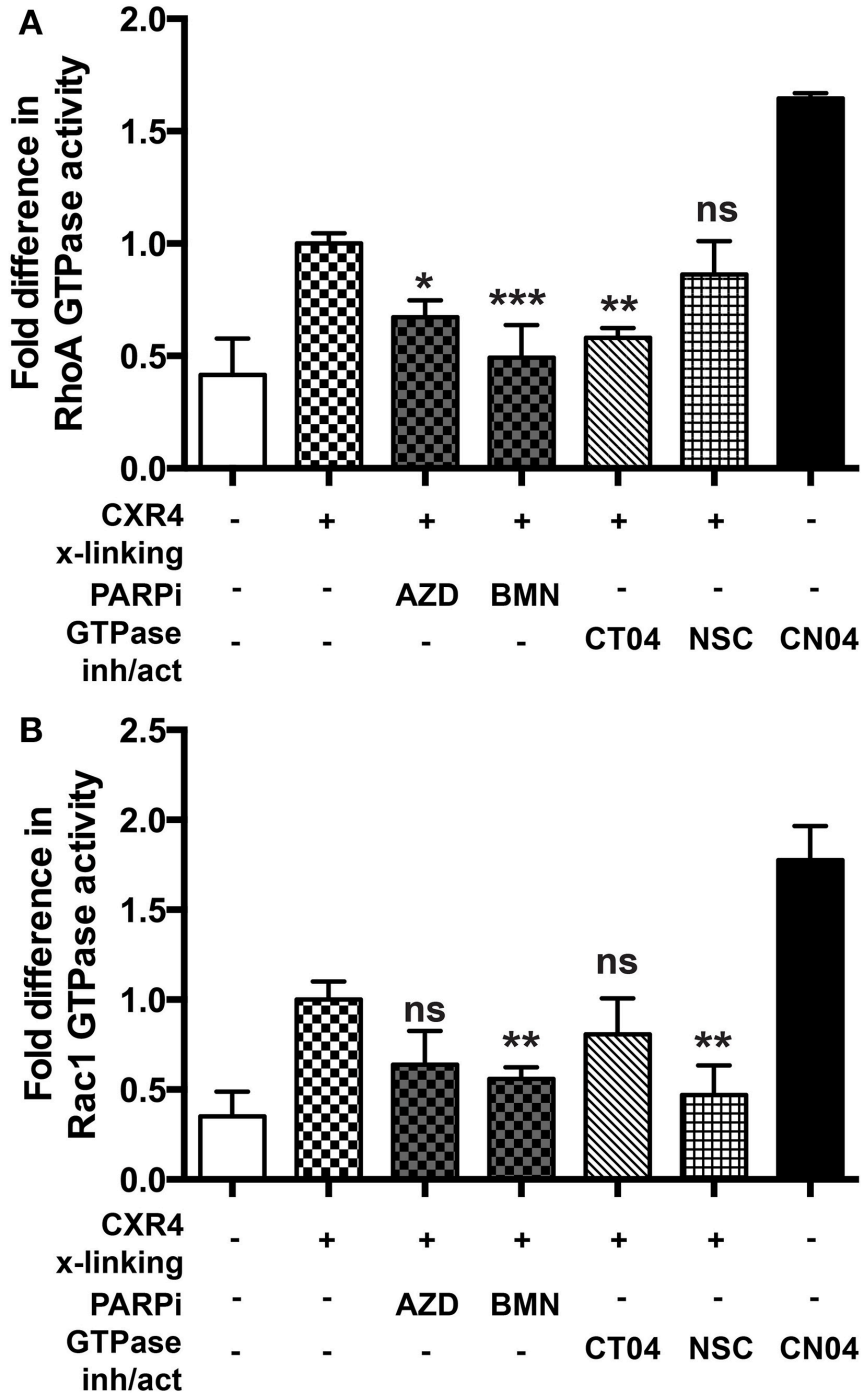
**PARP inhibition decreases Rac1 activation in primary MDM. (A)** RhoA and **(B)** Rac1 GTPases activity was measured in primary MDMs. GTPases were activated by cross-linking (x-linking) of the CXCR4 receptor with α-CXCR4 antibody (mimicking HIV-1 engagement with macrophage cells). The level of RhoA or Rac1 activity in non-treated cross-linked cells was assigned a value of 1 to calculate the relative ratio of activation. The results are shown as the mean value ± SEM from three independent experiments. (^*^p < 0.05, ^**^p < 0.005, and ^***^p < 0.0001 vs. cross-linked but inhibitor untreated cells).

Since RhoA and Rac1 play critical roles in influencing the replication of HIV-1 (Wang et al., 2000; Stolp and Fackler, 2011; Costantino et al., 2012), we explored the possibility that PARPi would suppress their activity in MDM activated with relevant stimuli. To detect the active, GTP-bound form of RhoA or Rac1, we used a commercially available G-LISA kit (Cytoskeleton Inc.). Induction of integrin signaling (leading to GTPase activation) can be achieved by cross-linking with antibodies mimicking engagement by HIV-1. Cross-linking of CXCR4 in MDM induced 2.5- and 2.8-fold increase of RhoA and Rac1 activity, respectively (Figures 5A,B). Of note the negative control (non-immune IgG) had no effect on small GTPase activation. Treatment with the PARPi, AZD and BMN, resulted in 40 and 55% reduction in RhoA activation, respectively (Figure 5A). RhoA specific inhibitor, CT04, treatment led to 50 ± 3% decrease in RhoA activation. Rac1 activation was diminished by 40 ± 5% when pretreated with BMN. Rac1 specific inhibitor, NSC, treatment led to 51 ± 4% decrease in Rac1 activation (Figure 5B). In summary, we have shown that PARPi alters RhoA and Rac1 activation in MDM.

### Inactivation of PARP prevents dephosphorylation of inhibitory sites of cofilin

**Figure 6:**
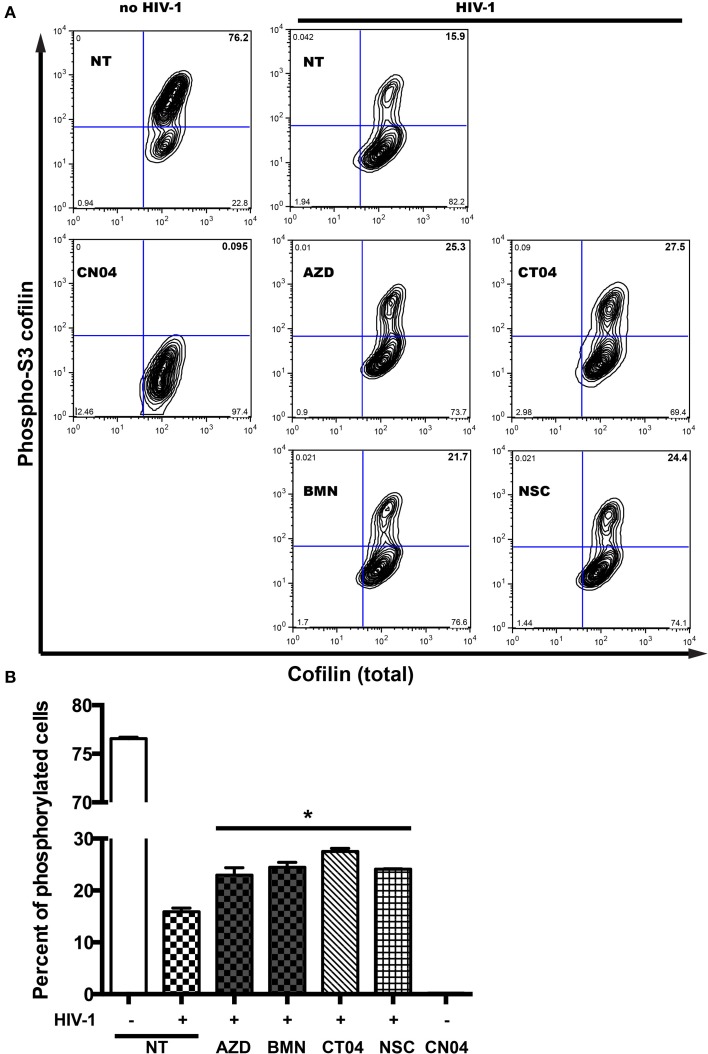
**PARP inhibition increases inhibitory site Ser3-cofilin phosphorylation. (A)** Representative contour plots from flow cytometry analysis of phospho-Ser3-cofilin and total cofilin in MDM stimulated by addition of HIV_ADA_ for 5 min. MDM were treated with or without PARPi for 1 h prior to HIV_ADA_ introduction. Rho and Rac GTPase inhibitors, CT04, NSC, and Rho/Rac activator, CN04, were used as controls. **(B)** Quantitation of phosphor-Ser3-cofilin cells in human MDM. The results are shown as the mean value ± SEM (^*^p < 0.05 vs. HIV-stimulated but inhibitor untreated cells) from three independent experiments utilizing MDM prepared from different donors.

Serial engagement of the primary viral receptor, CD4, and the co-receptor, CXCR4 or CCR5, are required for HIV entry into the cell (Maddon et al., 1986; Cocchi et al., 1995; Feng et al., 1996; Spear et al., 2012). This binding of HIV to respective receptors results in viral fusion, where the viral core is delivered into the cytoplasm. Furthermore, intracellular transport of the incoming genome toward the nucleus and of virions to budding sites requires functional interactions with the host actin cytoskeleton (Stolp and Fackler, 2011). Chemokine receptor signaling typically results in activation of Rho GTPases, which coordinate actin filament formation and cell motility, requiring efficient disassembly of preexisting actin filaments. A search for proteins that regulate filamentous and globular actin levels revealed an increase in the levels of an inactive phosphorylated form (phospho-serine 3) of the F-actin-severing protein, cofilin (Arber et al., 1998). After detection of cofilin phosphorylation in MDM without stimulation, we examined the dynamics of cofilin phosphorylation in cells undergoing changes in actin cytoskeletal reorganization during the first 5 min of HIV-1 contact (Costantino et al., 2012). HIV-1 interaction with MDM led to a 4.8-fold decrease in cofilin phosphorylation of the inhibitory site of serine 3 (Figure 6). As a positive control, we used the Rho GTPase activator, CN04, which resulted in total cofilin dephosphorylation of the inhibitory site (Figure 6). However, treatment of MDM with PARPi led to 13 and 16% increases in phosphorylation levels of the inhibitory site phospho-serine 3. The effects of RhoA and Rac1 specific inhibitors, CT04 and NSC, were similar to that of PARPi. Together, these results strongly suggest that PARP inhibition affects the actin-binding protein, cofilin, via RhoA and Rac1 suppression.

### PARP inhibition affects actin cytoskeleton rearrangements

**Figure 7:**
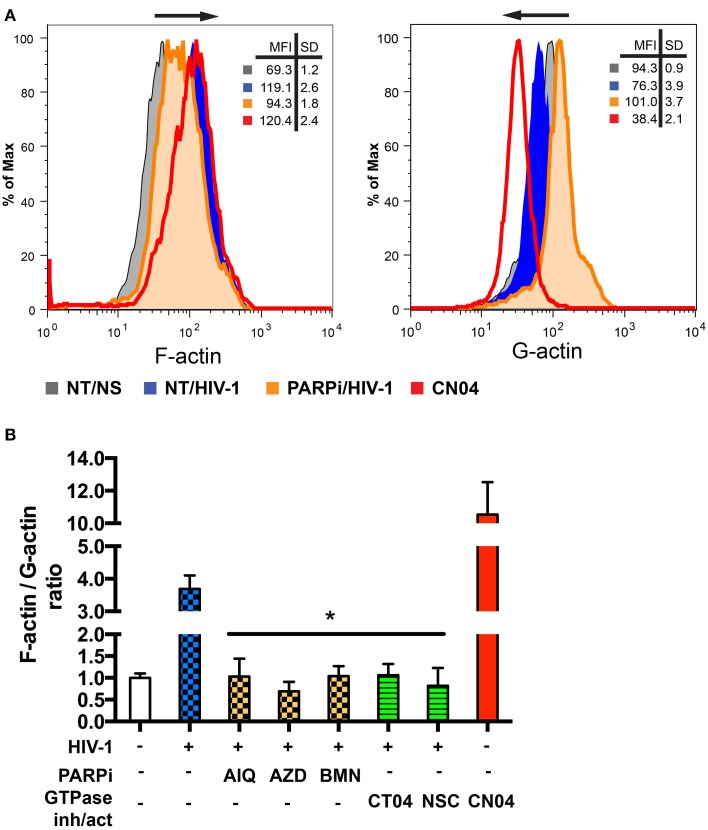
**PARP inhibitors suppress activation of small GTPases in MDM and affect actin rearrangements. (A)** Representative histograms from flow cytometry analysis of F-actin and G-actin in MDM stimulated by addition of HIV_ADA_ for 5 min. MDM were treated with or without PARPi for 1 h prior to HIV_ADA_ introduction. Rho and Rac GTPase inhibitors, CT04, NSC, and Rho/Rac activator, CN04, used as controls. **(B)** Graphical representation of F-actin to G-actin ratios. The results are shown as the mean value ± SEM (^*^p < 0.05 vs. HIV-stimulated but inhibitor untreated cells) from three independent experiments utilizing MDM prepared from different donors.

Actin rearrangements triggered by HIV-1 are regulated by severing cofilin, thus dissociating and facilitating actin filament depolymerization (Arber et al., 1998; Costantino et al., 2012). Since PARP inhibition affected dephosphorylation of the inhibitory site and thus activation of cofilin, we investigated actin rearrangements in MDM during the first 5 min of HIV-1 contact (Costantino et al., 2012) by flow cytometry. For comparison of F/G-actin ratios in MDM, F-actin was stained with phalloidin and G-actin was stained with DNAse I during HIV-1-adsorption to MDM with or without PARPi. F-actin staining increased upon contact with HIV-1, whereas G-actin staining decreased (Figure 7A). The Rho GTPase activator, CN04, which was used as a positive control, showed similar effects on F- and G-actin staining patterns (Figure 7A). MFI for F-actin and G-actin from different stimulations or treatments were used to calculate F/G-actin ratios. HIV-1 adsorption to MDM resulted in a 4-fold increase in F/G-actin ratio (Figure 7B). PARPi such as AIQ and BMN attenuated by 95 ± 3% and AZD completely blocked actin rearrangements in HIV-1-infected MDM (Figure 7B). The effects of RhoA and Rac1-specific inhibitors, CT04 and NSC, were similar to that of PARPi. Taken together, these results strongly indicate that PARP inhibition affects the actin cytoskeleton via RhoA and Rac1 inhibition.

### PARPi inhibit the activity of HIV-1 LTR

**Figure 8:**
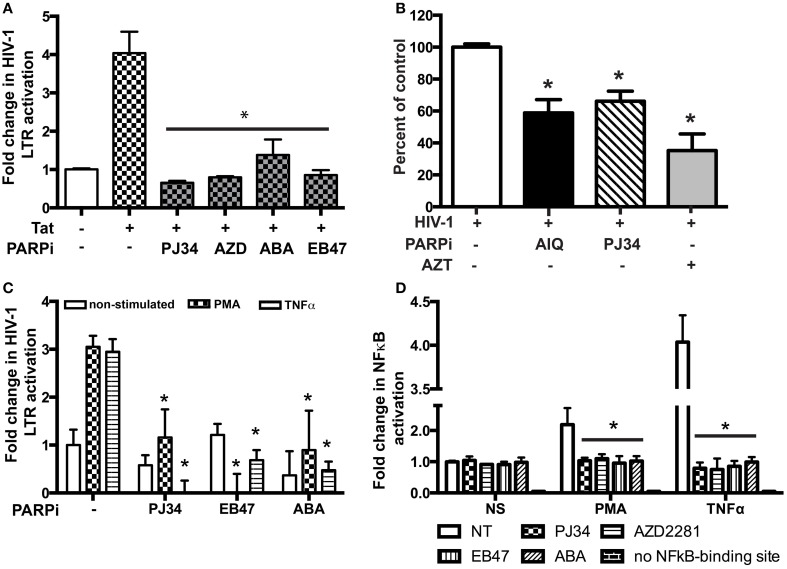
**PARP inhibitors suppress HIV-1 LTR activation in macrophages (MDM) by repressing NFkB activation. (A)** Tat-expressing plasmid and HIV-1 LTR-containing plasmid were used to transfect MDM with or without treatment with PARPi. Treatments were introduced 3 h post-transfection. **(B)** Single round HIV-1 infection of TZM-bl reporter cells pre-treated with PARPi or AZT. **(C)** HIV-1 LTR-containing plasmid was used to transfect MDM with or without treatment with PARPi. Treatments were introduced 1 h before PMA or TNFα. **(D)** Luciferase reporter plasmid (with/out NFkB-binding site) was used to transfect MDM with or without treatment with PARPi. The results shown are mean value ± SEM for quadruplicate cultures of MDM prepared from different donors (^*^p < 0.05 inhibitor untreated cells vs. stimulated cells in **A,C,D** or vs. HIV-1 infected cells in **B**).

To access the possibility that HIV-1 LTR activity is affected by inactivation of PARP, we performed single-round HIV-1 infection using the reporter line, TZM-bl cells. Pseudotyped HIV-1 virions, lacking the envelope protein, were produced and TZM-bl cells were infected as described in the Materials and Methods section. Since TZM-bl cells contain the β-gal reporter gene under the control of the HIV-1 LTR, we could measure its activity (Ramirez et al., 2013). The infection was allowed to continue for 4 h at which point the media containing virus was removed and changed to medium containing PARPi. AIQ treatment led to a 40 ± 5% reduction in β-gal-activity in HIV-1 infected TZM-bl cells as related to untreated (Figure 8B). PJ34 resulted in a 30 ± 3% inhibition. LTR acts as a switch in virus replication and can be triggered by agents such as Tat, TNFα, or PMA (Kameoka et al., 1999a,b; Rom et al., 2010). To further confirm the HIV-1 LTR inhibition, MDM were co-transfected with pEYFP-Tat101 plasmid or pYFP-C1 (control) in the presence or absence of PARPi and 24 h later analyzed for luciferase activity. Tat protein is a transactivating factor comprised of 86–101 amino acids encoded by HIV-1. The importance of HIV-1 Tat in the viral replication cycle and in the pathogenesis of AIDS is well documented (Rom et al., 2011). Indeed, HIV-1 Tat increased HIV-1 LTR activity by 4.2-fold, which was attenuated by PARPi application. The PARPi, ABA, reduced HIV-1 LTR activation by 65%, while PJ34, AZD, and EB47 completely abolished HIV-1 LTR activity (Figure 8A). Alternatively, MDM were transfected with HIV-1 LTR-luciferase reporter plasmid and then stimulated with TNFα or PMA for 24 h. Both TNFα and PMA stimulation led to 3-fold increases in HIV-1 LTR activity, which was entirely abrogated by selective PARPi inhibition (Figure 8C). Various host transcription factors have been shown to bind to and activate the LTR of HIV-1; NFκB protein was one of them (Kaufman et al., 1992). We and others have described PARP to be a NFκB co-activator in different types of cells during inflammatory processes and cancer (Kameoka et al., 2000; Hassa and Hottiger, 2002; Hauser et al., 2006; Liu et al., 2012; Nakagawa et al., 2015; Rom et al., 2015). To prove a connection of PARP inhibition to NFκB activation in macrophages, we used a luciferase reporter system where only the active form of NFκB would be able to turn on the promoter. Transfected cells were stimulated with TNFα and PMA in the absence or presence of PARPi and the medium was collected for reporter assay. Stimulation with TNFα resulted in a 2.1-fold increase in NFκB activation, whereas PMA led to a 4.2-fold up-regulation (Figure 8D). PARPi attenuated NFκB activation by 85–89% and up to 95%, in PMA- and TNFα-stimulated MDM, respectively (Figure 8D). Our results indicate that inhibitors of PARP decrease viral replication by antagonizing the HIV-1 LTR via disruption in NFκB signaling.

## Discussion

HIV-1 infection of macrophages plays an important role in the development of HAND (Kaul and Lipton, 2006). Production of pro-inflammatory molecules (like CCL2, CD40L, IP-10, IL-8) by HIV-1 infected macrophages is associated with HAND progression (Letendre et al., 2011). Therefore, approaches curtailing both virus replication and limiting inflammatory responses are necessary to prevent neuro-cognitive decline seen in HIV-1 infection. Our previous work demonstrated anti-inflammatory and barrier protecting effects of PARP inhibition in brain endothelial cells (Rom et al., 2015) and macrophages (unpublished observations). Here, we report efficient suppression of HIV-1 replication in human macrophages by PARPi and several potential mechanisms underlying this phenomenon.

There are few reports suggesting involvement of PARP in HIV-1 replication. PARP was required for efficient HIV-1 integration in murine PARP-1 knock out embryonic fibroblasts (Ha et al., 2001). PARP inactivation was shown to play a role in the HIV-1 life cycle, where PARP contributed to HIV-1 LTR function in U1 cells (Kameoka et al., 1999b, 2005) and competed for binding to TAR RNA with p-TEFb in *in vitro* gel retardation studies (Parent et al., 2005).

The involvement of PARP in HIV-1 gene expression seems to be complicated depending on the inhibitors used in the study and/or the system used, including cells and inducers. Our results revealed that PARPi diminished HIV-1 replication in primary human MDM, whether applied prior to or after infection. No HIV-1 strain specificity was noted. Post-infection addition of PARPi, such as AIQ, AZD, and PJ34, had a much stronger effect on HIV-1 infection, than when inhibitors were added to MDM before infection, whereas EB47 had the same effects on HIV-1 infection. Interestingly, BMN had a significant effect only in pretreatment and not after. There is a possibility that different compounds have diverse kinetics in absorption as well as in retention in the cell.

**Figure 9:**
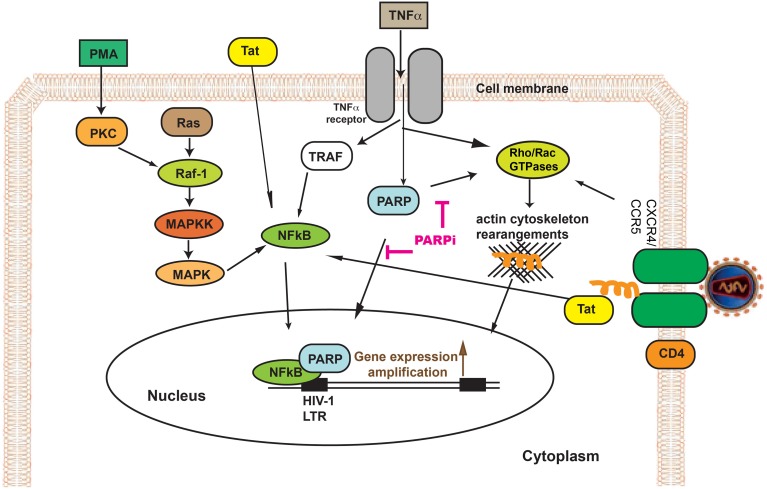
**Effect of PARP inhibition on HIV-1 infection and replication**.

HIV-1 infection could be diminished at different steps (interactions with receptors, receptor internalization, cytoskeleton rearrangement, and efficient integration). HIV-1 entry into target cells requires serial engagement of the primary viral receptor, CD4, and co-receptor, CXCR4 or CCR5 (Maddon et al., 1986; Cocchi et al., 1995; Feng et al., 1996; Spear et al., 2012). HIV binding, receptor clustering and intracellular transport of the viral genome toward the nucleus as well as to budding sites happens to necessitate functional interactions with the host actin cytoskeleton (Stolp and Fackler, 2011). It has been shown in CD4+ lymphocytes that suppression of cytoskeleton regulators (Arp2/3 and WAVE2) prevented HIV-1 infection diminishing its intracellular migration (Spear and Wu, 2014). Our data indicate that PARPi prevented HIV-1 from hijacking the cytoskeletal cell compartment, shown by complete blockage of F/G-actin transition by preventing the actin severing protein, cofilin, to become activated via attenuation of RhoA/Rac1 signaling (Figure 9).

In addition, PARP, being a co-activator of NFκB (Kameoka et al., 1999b, 2005), prevented HIV-1 gene transcription by obstructing HIV-1 LTR activation (Figure 9). The transcription factor NF-κB plays a critical role in immune and inflammatory responses. HIV-1 is known to produce inflammatory responses in wide variety of cells in different organs: brain, lungs, intestine, and kidneys, etc. The anti-inflammatory effects of PARP have been demonstrated in numerous *in vivo* and *in vitro* studies (Kraus and Lis, 2003; Erdélyi et al., 2005; Liu et al., 2012), some of which are due to effects of PARP on NF-κB function (Zerfaoui et al., 2009, 2010; Swindall et al., 2013).

Several PARP inhibitors are at different stages of cancer treatment clinical trials (Cavone et al., 2011) including two used in our study. Talazoparib (BMN) and Olaparib (AZD) showed suppressive effects on HIV-1 replication. PARPi might increase the therapeutic potential of currently used anti-retroviral treatments to reduce peripheral viral load. In addition to anti-retroviral potential of PARPi, the anti-inflammatory effects of PARP suppression in rodent models of meningitis and stroke, brain injury, and MS are important for HAND treatment as they attenuate leukocyte infiltration across the blood brain barrier and neuroinflammation (Lenzsér et al., 2007; Farez et al., 2009; Lescot et al., 2010; Rom et al., 2015). Approval of PARPi for clinical trials by the FDA assures fast translation of PARPi for therapy of immune/inflammatory disorders, HAND and other HIV-associated end-organ dysfunctions.

## Author contributions

Concept and design of the experiments, interpretation of results (SR, YP), data acquisition and analysis (SR, HD, NR). All authors participated in drafting, revising and final approval of the manuscript, and agree to be accountable for all aspects of the work.

### Conflict of interest statement

The authors declare that the research was conducted in the absence of any commercial or financial relationships that could be construed as a potential conflict of interest.

## Abbreviations

PARP: Poly(ADP-ribose) polymerase
NFκB: nuclear factor kappa-light-chain-enhancer of activated B cells
ABA: 3-aminobenzamide
AZD: AZD2281 (Olafarib)
BMN: BMN-673 (Talazoparib)
AIQ-5: aminoisoquinolinone
PJ34: [N-(6-oxo-5,6-dihydro-phenanthridin-2-yl)-N,N-dimethylacetamide hydrochloride
EB47: 5′-deoxy-5′-[4-[2-[(2,3-dihydro-1-oxo-1H-isoindol-4-yl)amino]-2-oxoethyl]-1-piperazinyl]-5′-oxoadenosine dihydrochloride
MDM: monocyte-derived macrophages
PMA: phorbol 12-myristate 13-acetate
FACS: fluorescence-activated cell sorting
MFI: mean fluorescence intensity.
