# Supplementary material for: The dual action of poly(ADP-ribose) polymerase -1 (PARP-1) inhibition in HIV-1 infection: HIV-1 LTR inhibition and diminution in Rho GTPase activity
Source: Front Microbiol. 2015 Aug 25;6:878. doi: 10.3389/fmicb.2015.00878 (PMC4548080; doi:10.3389/fmicb.2015.00878)

The dual action of poly(ADP-ribose) polymerase -1 (PARP-1) inhibition in HIV-1 infection: HIV-1 LTR inhibition and diminution in Rho GTPase activity

Slava Rom, Nancy L. Reichenbach, Holly Dykstra and Yuri Persidsky

Supplemental Figure 1. *PARP inhibition does not affect MDM viability.* The Live-Dead assay was performed on PARPi-treated MDM. The number of non-treated cells was assigned as 100%. Saponin, a cell death initiator, was also included as a control. The results shown are mean values ± SEM for duplicate cultures of MDM prepared from different donors (*p<0.05 saponin-treated cells versus non-treated cells).

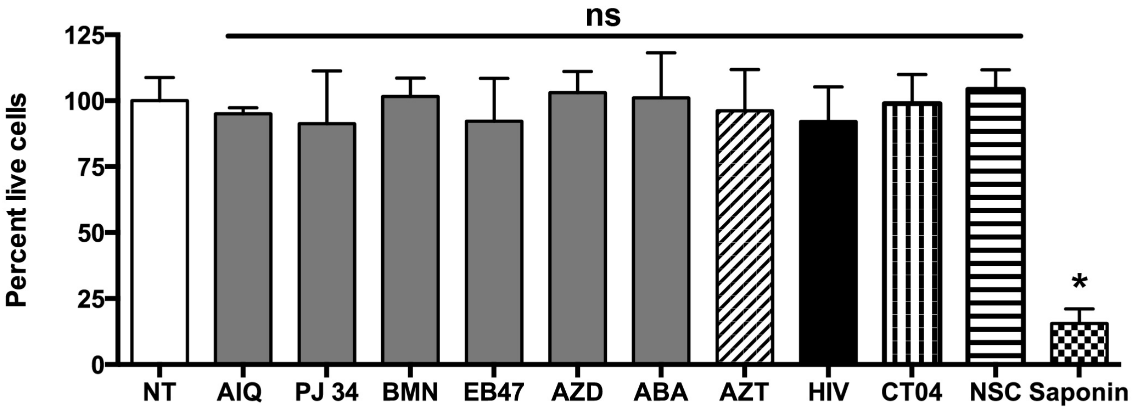

Supplement: Supplementary file 1 [file Image1.PDF]
